# Study protocol of associated criteria used in investigating septic transfusion reactions (STRs): A scoping review about available evidence

**DOI:** 10.1371/journal.pone.0262765

**Published:** 2022-01-20

**Authors:** Dilaram Acharya, Antoine Lewin, Amaury Gaussen, Gilles Lambert, Christian Renaud, Karlitaj Nawej, Thomas G. Poder

**Affiliations:** 1 School of Public Health, Université de Montréal, Montréal, Québec, Canada; 2 Medical Affairs and Innovation, Héma-Québec, Montréal, Québec, Canada; 3 Faculty of Medicine and Health Science, Université de Sherbrooke, Sherbrooke, Québec, Canada; 4 Direction régionale de santé publique, Centre intégré universitaire de santé et de services sociaux du Centre-Sud-de-l’Île-de-Montréal du Québec, Montréal, Québec, Canada; 5 Direction des risques biologiques et de la santé au travail, Institut national de santé publique du Québec, Montréal, Québec, Canada; 6 Centre de Recherche de l’Institut Universitaire en Santé Mentale de Montréal, CIUSSS de l’Est-de-l’île-de-Montréal, Montréal, Canada; Qatar University, QATAR

## Abstract

**Background and objective:**

Assessment criteria for septic transfusion reactions (STRs) are variable around the world. A scoping review will be carried out to find out, explore and map existing literature on STRs associated criteria.

**Methods:**

This scoping review will include indexed and grey literatures available in English or French language from January 1, 2000, to December 31, 2021. Literature search will be conducted using four electronic databases (i.e., MEDLINE *via* PubMed, Web of Science, Science Direct, and Embase *via* Ovid), and grey literatures accompanying the research questions and objectives. Based on the inclusion criteria, studies will be independently screened by two reviewers for title, abstract, and full text. Extracted data will be presented in tabular form followed by a narrative description of inputs corresponding to research objectives and questions.

## Introduction

Septic transfusion reactions (STRs) resulting from the bacterial contaminated blood components transfusion remain a significant cause of transfusion associated morbidities and mortalities, despite having prevention and control measures in place such as pathogen reduction, and mandatory post collection culturing of platelets [1–3]. Bacterial contamination of blood or blood components may occur as a result of donor bacteremia, contamination during blood collection, contamination of the collection pack, and finally contamination during the blood processing procedure [4].

During the 90s, bacterial contamination of blood components was recognized as a major cause of transfusion-transmitted infection, accounting for between 14% and 24% of transfusion-associated fatalities reported to the US Food and Drug Administration (FDA) [5, 6]. Although all types of blood components have been reported with contamination leading to infections, platelets (PLTs) stored at room temperature, allowing growth of many bacterial organisms, seem most frequently involved in STRs [7]. A recent report from the US FDA reported that between 2012 and 2016, 18 fatalities were attributed to bacterial contamination of red blood cells (n = 7), pooled platelets (n = 2), apheresis platelets (n = 8), and plasma (n = 1) [8], while nearly 21 million of blood components were transfused each year in the United-States [9]. Importantly, STRs are underdiagnosed when blood recipients are on broad-spectrum antibiotics or have other underlying medical conditions with overlapping signs and symptoms [10–12].

Continuous improvement and implementation of bacterial STRs reduction include a wide-range of strategies both from blood donor to receiver perspectives such as donor screening, skin disinfection, diversion of initial blood collection, pre-storage culturing, post transfusion culturing of blood recipients, and blood components, and additionally, there is also provision of ongoing education, and up-to-date information regarding STRs to the related stakeholders [13–15]. Despite having implemented preventive and control measures in the reduction of transfusion-transmitted septic reactions (TTSRs) through the use of existing hemovigilance system in many countries worldwide [16–18], there are still reports of TTSRs. For instances, a recent peer-reviewed publication including hemovigilance data from North America, Europe, Africa, and Oceania, reported that the transfusion-transmitted bacterial infection frequency ranged 1:14,515 to 1:384,903 in transfused platelets, and 1:96,850 to 1:3,448,275 in transfused erythrocytes in 2016 and 2017 [19]. In Canada, according to the Transfusion Transmitted Injuries Surveillance System (TTISS), bacterial infection contributed to a total of 0.8% (33/3957) of adverse reactions following blood components and plasma derivative transfusions during the period of 2006–2012 [20]. Investigating TTSRs and associated suspected risks is difficult as blood culture is usually performed on passive reporting of suspected acute transfusion reactions when there is no recognizable signs and symptoms of infection. In addition, there are reports of high rate of false positive culture results related to STRs [21] and variability of criteria used in the identification of those STRs [22].

Although less transfusion-transmitted bacterial infections are reported these days, identification of STRs and provision of safe blood products are still ongoing challenge in transfusion medicine. Given that there is no consensus in a set of criteria associated with STRs in the published and grey literatures, it is imperative to answer the specific research questions that will further assist identification of research gap and help identify more precise criteria associated with STRs.

With these backgrounds, the aims and objectives of this scoping review are to find out, explore and map the existing literature on STRs associated criteria. Based on the country-specific preventive and control measures implemented to mitigate STRs, this review aims to: (1) report criteria used for STRs detection in published and grey literatures; and (2) identify the prevalence of STRs related to platelets, red blood cells and plasma among those selected literatures.

It is expected that the results of this review will inform policy makers, planners, researchers, and governments in transfusion medicine, and help identify the gap for further studies.

## Review protocol

This scoping review protocol will review the published and grey literatures systematically for all STRs and associated criteria. The proposed review will follow the methodological framework recommended by Peters et al. [23], such as: 1) Defining and aligning the objective/s and question/s, 2) Developing and aligning the inclusion criteria with the objective/s and question/s, 3) Describing the planned approach to evidence searching, selection, data extraction, and presentation of the evidence, 4) Data extraction, 5) Presentation of the results, 6) Study implications and dissemination. This scoping review will follow the quality appraisal of the selected literatures. Quality assessment will follow the identification of the key characteristics of the studies such as the appropriateness of the study design to the research question being asked, the suitability of the sample, the methods used to recruit the sample, the methods used to obtain the results, and general direction of the study findings. More importantly, examining the possible reasons for study similarities or differences. Since this manuscript is a scoping review protocol, it does not require ethical approval letter from an institutional review board (IRB) or ethical committee and therefore, ethical approval for this protocol will automatically be waived.

### Objectives

The main research question is: “What are the criteria associated with STRs that are reported on published and grey literatures?”

Based on the country-specific preventive and control measures implemented to mitigate STRs, the specific research questions derived from the general research question are:

1. What are the different criteria used for STRs suspicions and their sensitivity and specificity in published and grey literatures?
2. What is the prevalence of STRs related to different blood components (Platelets, Red Blood Cells (RBCs), and Plasma) reported?

## Methods

### Eligibility criteria

#### Developing and aligning the inclusion criteria with the objective/s and question/s

This study will follow the population, concept, and context (PCC) as recommended in the Joanna Briggs Institute (JBI) guideline. The inclusion criteria of this review should meet the followings:

- Population receiving blood transfusion (platelet, RBC and/or plasma).
- Concept will be all those individual and collective criteria used to identify situation to be investigated for TRSRs.
- Context will be settings such as the health care, hemovigilance, and regional and provincial health care service facilities.
- Scope: quantitative and qualitative studies, both in published and grey literatures, diffused between January 1, 2000, to December 31, 2021, in English or French language, for which full-text articles or reports are available/obtained

### Exclusion criteria

The study will have the following exclusion criteria: duplicate publications, study has no outcome of interest included, non-original research, including editorials, opinion pieces, letters, protocols, studies published before January 1, 2000, and those that are in languages other than English or French language.

#### Search method

The literature search will be included to all published peer-reviewed articles and all relevant guidelines and grey literature pertaining to the research objectives and questions in the English or French languages. For all peer-reviewed published articles, a search will be performed using the following electronic databases: MEDLINE *via* PubMed, Web of Science, Science Direct, and Embase *via* Ovid, while for those in grey literature, we will use conference proceedings, theses and dissertations, association reports, government reports (e.g., federal, provincial, and regional, from organization’s websites), and WorldCat.

We will conduct an initial search using key words and controlled vocabulary for peer-reviewed articles, and grey literature. We have demonstrated examples of our search strategy including our search terms and queries in different databases (S1 and S2 Tables in S1 Appendix). The search results will be compiled, and duplicates will be removed using EndNote (Clarivate Analytics, Philadelphia, United States). An additional literature search will be conducted by hand search in key journals (i.e., Transfusion, Vox Sanguinis, Blood, Critical Care Medicine, Clinical Infectious Diseases, Transfusion Clinique et Biologique, Transfusion Medicine Reviews, Blood Transfusion, BMC Hematology, Journal of Blood Medicine, Journal of Hematology, Blood Reviews, Journal of Thrombosis and Haemostasias), and from the reference list of all included studies to identify other studies not captured through the electronic search.

#### Study selection process and review management

**Fig 1:**
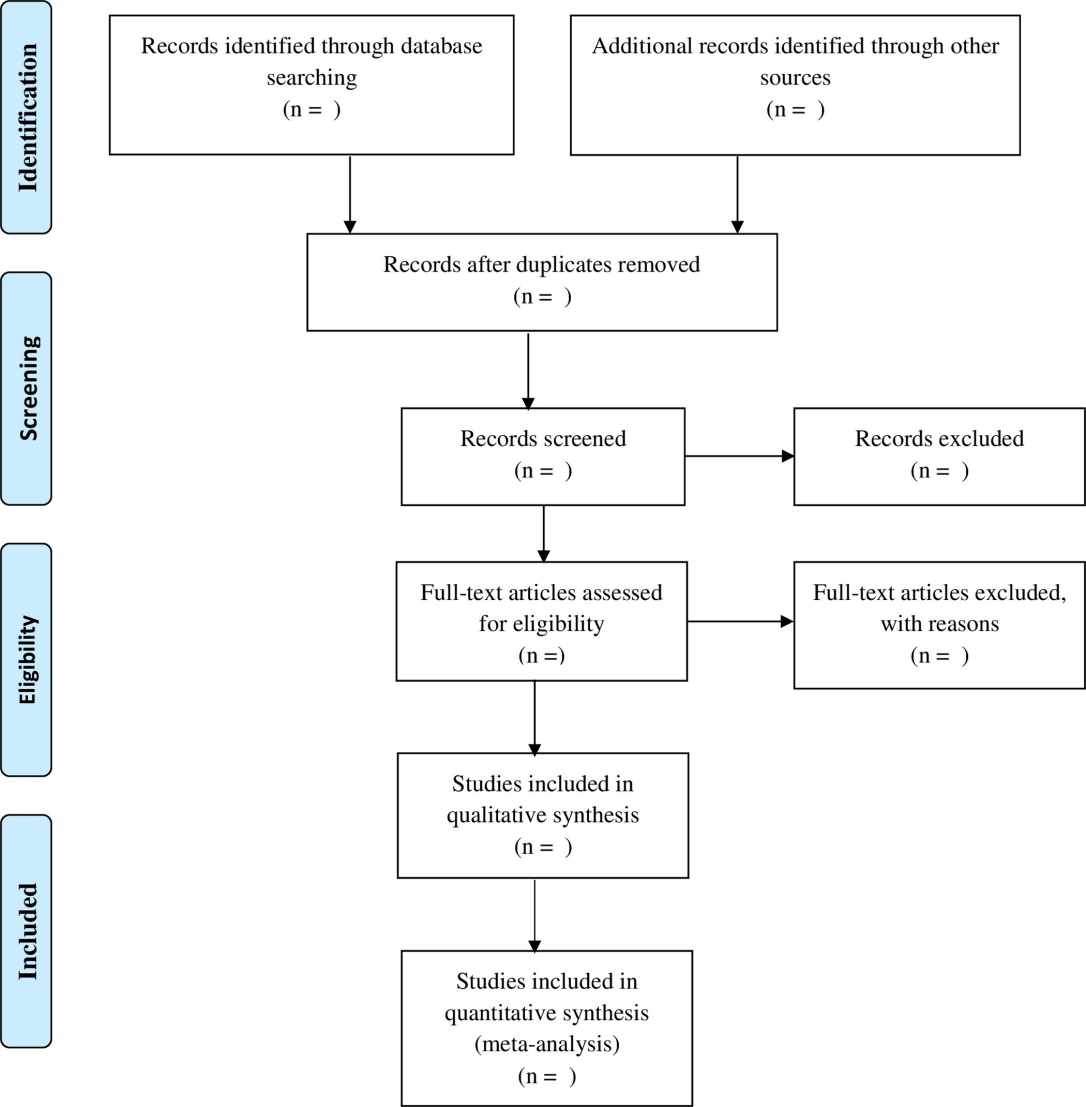
PRISMA flowchart for the selection of eligible studies.

All published and grey literatures will be sought in English or French languages from January 1, 2000, to December 31, 2021, for which full text are available with an up-to-date information, while non-original research, including editorials, opinion pieces, letters, and protocols, will be excluded from the study. All available and selected literature will be complied following the Preferred Reporting for Systematic Review and Meta-analysis-scoping review (PRISMA-SCR) [24]. The authors will be divided into groups of two and will independently conduct the selection of full text articles and other selected literatures to sort out their eligibility for inclusions based on the articles through title and abstract analysis. In cases of disagreements, consensus will be made based on mutual agreement and obtention of opinion from experts in the field. Endnote software will be made use for the management of the results of the search. The PRISMA flowchart will be used to describe the selection procedure (Fig 1).

#### Data extraction

The data charting will be made in a logical order and a descriptive summary of the results will be presented in line with the research questions and objectives. The tabular form will be used to record the key information such as author/s, year of publication, country of origin, aims, study design, sample selection, population and sample size, level of evidence, data quality, reference, results, etc. related to the review questions. More specifically, among others, extracted data will include the following information: 1) definition of STR mentioned, 2) sign and symptoms of STRs, 3) defining body for STR (e.g., international, national, provincial, regional), 4) type of blood components, 5) fatality (yes vs no), 6) prevalence rates of STR for each blood components, 7) sensitivity and specificity of STR criteria.

#### Presentation of the results

The results will be presented in tabular form along with a narrative summary aligned with the research questions of the scoping review. In the table, data will be grouped as per the study type. Descriptive analysis will be conducted to summarize the information obtained in the form of rates, mean and standard deviation wherever necessary. The results will be discussed in line with the future work, and practice in Canadian and global context.

#### Study implications and dissemination

This scoping review has the potential to advise policy makers, health care providers and researchers on how possible non-immunological septic transfusions are assessed, and thus, might be the milestone in the development of a guideline for assessment and management of STRs in Canadian and global context. The scoping review will be made available to public though peer-reviewed publications and public-oriented media types.

## Supporting information

S1 Appendix Search summary plan and literature search strategy. (DOCX)
