# Supplementary material for: Study protocol of associated criteria used in investigating septic transfusion reactions (STRs): A scoping review about available evidence
Source: PLoS One. 2022 Jan 20;17(1):e0262765. doi: 10.1371/journal.pone.0262765 (PMC8775533; doi:10.1371/journal.pone.0262765)
Supplement: S1 Appendix — (DOCX) [file pone.0262765.s001.docx]

**S1 Appendix: search summary plan and literature search strategy**

**Table 1 Timeline for updating the search results**

| **Sources** | **Results (with duplicates) – baseline + update** | **Baseline Results (unique)**  **(A)**  **August 1, 2021, to October 10** | **Update Results (unique)**  **(B)**  **October 11, 2021, to November 10, 2021** | **Update Results (unique)**  **(C)**  **November 11, 2021, to December 31, 2021** | **Update A, B and C Results (unique)**  **(A+B+C)**  **November 11, 2021, to December 31, 2021** |
| --- | --- | --- | --- | --- | --- |
| PubMed | - | A | B | C | A+B+C |
| Ovid Embase | - | A | B | C | A+B+C |
| Science Direct | - | A | B | C | A+B+C |
| Web of Science | - | A | B | C | A+B+C |
| Hand search to key Journals | - | A | B | C | A+B+C |
| WorldCat | - | A | B | C | A+B+C |
| AABB | - | A | B | C | A+B+C |
| PHAC | - | A | B | C | A+B+C |
| SJCRH | - | A | B | C | A+B+C |
| ARC | - | A | B | C | A+B+C |
| CDC | - | A | B | C | A+B+C |
| Canadian Blood Services | - | A | B | C | A+B+C |
| Others | - | A | B | C | A+B+C |

AABB, the American Association of Blood Banks; PHAC, Public Health Agency of Canada; SJCRH, St. Jude Children’s Research Hospital; ARC, the American Red Cross; CDC, Centers for Disease Control and Prevention.

**We will use the following databases with their respective search terms. We limited our search to English or French language for a period of January 1, 2000, to December 31, 2021.**

**Table 2 Detail strategic plan for literature search**

| **Databases (Electronic-Primary articles)** | **Search strategy** |
| --- | --- |
| MEDLINE (via PubMed) | #1 Search: ((transfusion reaction[MeSH Terms]) AND ((bacterial infections[MeSH Terms]) OR (red blood cells[MeSH Terms]) OR (erythrocytes[MeSH Terms]) OR (plasma*[MeSH Terms]) OR (blood platelets[MeSH Terms]) OR (blood transfusion[MeSH Terms]) OR (blood component transfusion[MeSH Terms]) OR (platelet transfusion[MeSH Terms]) OR (plateletphereses[MeSH Terms]) OR (blood plasma[MeSH Terms]) OR (shock, septic[MeSH Terms])) AND (humans[MeSH Terms]))  #2 Search: ((transfusion transmitted AND (infection OR bacterial)) OR "septic transfusion") AND ((assessment OR criteria OR standard OR diagnostic OR diagnosis OR definition OR harmonized OR consensus OR detection OR monitoring OR recognition OR hours OR temperature OR presentation OR signs OR symptoms) OR "passive surveillance") AND (reaction OR contamination OR infection)  #3 Search: #1 AND #2 Filters: Clinical Trial, Journal Article, Randomized Controlled Trial, Humans  #4 Search: (((transfusion transmitted [Title/Abstract]) AND ((infection [Title/Abstract]) OR (bacterial [Title/Abstract]))) OR ("septic transfusion"[Title/Abstract])) AND ((reaction[Title/Abstract]) OR (contamination[Title/Abstract]) OR (infection[Title/Abstract]))  #5 Search: (((assessment[Text Word]) OR (criteria[Text Word]) OR (standard[Text Word]) OR (diagnos*[Text Word]) OR (definition[Text Word]) OR (harmonize*[Text Word]) OR (consensus[Text Word]) OR (detection[Text Word]) OR (monitoring[Text Word]) OR (recognition[Text Word]) OR (hours[Text Word]) OR (temperature[Text Word]) OR (presentation[Text Word]) OR (signs[Text Word]) OR (symptoms[Text Word])) OR (passive surveillance[Text Word])) AND ((reaction[Text Word]) OR (contamination[Text Word]) OR (infection[Text Word]))  #6 Search: #4 AND #5  #7 Search: #3 OR #6  # 8 Search: #1 AND #6  #9 Search: #8 OR #9  #10 Search: #4 AND #5  #11 Search: #1 AND #12 |
| Embase (*via* Ovid) | #1 ((exp "transfusion reaction"/) AND ((exp "bacterial infections"/) OR (exp "red blood cells"/) OR (exp erythrocytes/) OR (exp plasma*/) OR (exp "blood platelets"/) OR (exp "blood transfusion"/) OR (exp "blood component transfusion"/) OR (exp "platelet transfusion"/) OR (exp plateletphereses/) OR (exp "blood plasma"/) OR (exp "shock, septic"/)) AND (exp humans/)) "{Including Limited Related Terms}"  #2 ((("transfusion transmitted" AND (infection OR bacterial)) OR "septic transfusion" ) AND (assessment OR criteria OR standard OR diagnostic OR diagnosis OR definition OR harmonized OR consensus OR detection OR monitoring OR recognition OR hours OR temperature OR presentation OR signs OR symptoms OR "passive surveillance" ) AND (reaction OR contamination OR infection )).mp.  "#3 1" AND (#2 ((("transfusion transmitted" AND (infection OR bacterial)) OR "septic transfusion" ) AND (assessment OR criteria OR standard OR diagnostic OR diagnosis OR definition OR harmonized OR consensus OR detection OR monitoring OR recognition OR hours OR temperature OR presentation OR signs OR symptoms OR "passive surveillance" ) AND (reaction OR contamination OR infection )).mp.)  #4 ((("transfusion transmitted" AND (infection OR bacterial)) OR "septic transfusion”) AND (reaction OR contamination OR infection )).tw.  #5 ((assessment OR criteria OR standard OR diagnos* OR definition OR harmonize* OR consensus OR detection OR monitoring OR recognition OR hours OR temperature OR presentation OR signs OR symptoms OR "passive surveillance”) AND (reaction OR contamination OR infection)).mp.  "#6 4" AND (#5 ((assessment OR criteria OR standard OR diagnos* OR definition OR harmonize* OR consensus OR detection OR monitoring OR recognition OR hours OR temperature OR presentation OR signs OR symptoms OR "passive surveillance”) AND (reaction OR contamination OR infection)).mp.) |
| Science Direct | (assessment OR criteria OR standard OR diagnosis OR definition OR harmonized OR consensus OR detection OR presentation) ((transfusion transmitted AND (infection OR bacterial)) OR "septic transfusion") AND (reaction OR contamination OR infection) |
| Web of Science | (AB= (((transfusion transmitted AND (infection OR bacterial)) OR "septic transfusion") AND (reaction OR contamination OR infection))) AND ALL= (((assessment OR criteria OR standard OR diagnostic OR diagnosis OR definition OR harmonized OR consensus OR detection OR monitoring OR recognition OR hours OR temperature OR presentation OR signs OR symptoms) OR "passive surveillance") ) |
| Hand search to key Journals  (Transfusion; Vox Sanguinis; Blood; Critical Care Medicine; Clinical Infectious Diseases; Transfusion Clinique et Biologique; transfusion medicine reviews; blood transfusion; BMC hematology; Journal of blood medicine; Journal of hematology; Blood reviews; Journal of Thrombosis and Haemostasias) | (Assessment OR criteria OR standard OR diagnosis OR definition OR harmonized OR consensus OR detection OR presentation) ((transfusion transmitted AND (infection OR bacterial)) OR "septic transfusion") AND (reaction OR contamination OR infection) |
| **Databases (grey literatures)** | |
| WorldCat | (assessment OR criteria OR standard OR diagnosis OR definition OR harmonized OR consensus OR detection OR presentation) ((transfusion transmitted AND (infection OR bacterial)) OR "septic transfusion") AND (reaction OR contamination OR infection) |
| AABB | (assessment OR criteria OR standard OR diagnosis OR definition OR harmonized OR consensus OR detection OR presentation) ((transfusion transmitted AND (infection OR bacterial)) OR "septic transfusion") AND (reaction OR contamination OR infection) |
| PHAC | (assessment OR criteria OR standard OR diagnosis OR definition OR harmonized OR consensus OR detection OR presentation) ((transfusion transmitted AND (infection OR bacterial)) OR "septic transfusion") AND (reaction OR contamination OR infection) |
| SJCRH | (assessment OR criteria OR standard OR diagnosis OR definition OR harmonized OR consensus OR detection OR presentation) ((transfusion transmitted AND (infection OR bacterial)) OR "septic transfusion") AND (reaction OR contamination OR infection) |
| ARC | (assessment OR criteria OR standard OR diagnosis OR definition OR harmonized OR consensus OR detection OR presentation) ((transfusion transmitted AND (infection OR bacterial)) OR "septic transfusion") AND (reaction OR contamination OR infection) |
| CDC | (assessment OR criteria OR standard OR diagnosis OR definition OR harmonized OR consensus OR detection OR presentation) ((transfusion transmitted AND (infection OR bacterial)) OR "septic transfusion") AND (reaction OR contamination OR infection) |
| Canadian Blood Services | (Assessment OR criteria OR standard OR diagnosis OR definition OR harmonized OR consensus OR detection OR presentation) ((transfusion transmitted AND (infection OR bacterial)) OR "septic transfusion") AND (reaction OR contamination OR infection) |
| Others | (Assessment OR criteria OR standard OR diagnosis OR definition OR harmonized OR consensus OR detection OR presentation) ((transfusion transmitted AND (infection OR bacterial)) OR "septic transfusion") AND (reaction OR contamination OR infection) |

AABB, the American Association of Blood Banks; PHAC, Public Health Agency of Canada; SJCRH, St. Jude Children’s Research Hospital; ARC, the American Red Cross; CDC, Centers for Disease Control and Prevention.
